# Educational constraints on marital sorting and income mobility

**DOI:** 10.1371/journal.pone.0313710

**Published:** 2025-02-19

**Authors:** Nan Gao

**Affiliations:** School of Economics, Anhui University, Hefei, Anhui, China; IIPS: International Institute for Population Sciences, INDIA

## Abstract

This paper examines the effect of educational assortative mating on overall income mobility in terms of both inter- and intragenerational mobility. In recent years, educational assortative mating has become a trend in China, and this marital sorting pattern not only contributes to marriage stability but is also related to increased income mobility. Educational assortative mating affects the distribution of educational resources among families with diffferent educational qualifications. Families with higher educational qualifications have more educational resources, thus obtaining a more solid income status. Educational assortative mating has the most significant influence on the income mobility of the low-income group, as high-quality educational resources are the key to enabling this group to raise its income status. This paper argues that, in the future, income mobility should be promoted by evenly distributing social education resources and reducing the resource constraints on the upward mobility of the low-income group.

## 1. Introduction

The marriage market is competitive. The marriage search is costly and uncertain, so young men and women choose their spouses quite carefully to maximize marriage utility [1]. When searching for a spouse on the marriage market, people tend to set a limit on their choice based on criteria such as family background, age, education, and occupation. They then determine their partner based on the principle of complementarity. Unlike factors such as occupation and temporary income, education level is often more stable and can be used to judge an individual’s ability to develop in the long term. It has thus become increasingly important as a mate selection criterion [2,3]. The theory of homogamy suggests that individuals of similar social class are more likely to enter into marriage, and that a similar set of values, lifestyle, and the acceptance of both parties’ social networks are conducive to promoting marital stability [4]. Education level, specifically, is a direct reflection of an individual’s cultural capital. Individuals with a similar level of education levels tend to have more of a common language, which makes their marriage easier [5].

“Marriage between the right families” is a deeply engrained principle in China’s marriage system. Although the selection criteria for marriage have changed constantly with time—from the class and rank of the marriageable man and woman to their education and ways of thinking—the concept has never been discarded from the marriage market [6]. With economic and social development, improved levels of education, and the popularization of female education, the demand for homogamy in the marriage market have become more open and liberalized. Young men and women care less about endowments such as the family conditions of their spouses and more about their earned status, such as the level of personal human capital [7]. With the increasing returns to education, people place higher hopes on finding a spouse with similar levels of education in their marital sorting decisions to maximize family returns [8,9]. Special matchmaking groups such as “Masters and Doctors Matching Group” and “Overseas Students Matching Group” have become active in China in recent years. An increasing number of Chinese residents are showing a greater inclination to seek spouses who possess a similar level of educational, which has led to the increasingly prominent phenomenon of educational assortative mating. “Matching between the right educational resources” has gradually become the key marital demand among Chinese residents in the new era [10,11].

Spouses’ educational level is directly related to family wealth, as is the integration of educational resources between spouses. Many studies, therefore, often link education-homogeneous marriage with social inequality [12,13]. At the same time, social inequality is linked with income mobility. Income mobility reflects the vertical adjustability of people’s income status, which can be further classified as intergenerational mobility and intragenerational mobility [14,15]. For Chinese citizens, their level of income mobility is related to their living conditions and social stability. Intergenerational mobility and intragenerational mobility reflect the degree to which social wealth is transferred between two generations or within the same generation, respectively. When people’s mobility increases, they can reap more social benefits, improve their life satisfaction, and enhance their subjective wellbeing [16,17]. Some studies have found that improved intergenerational mobility in China has increased the stability of the family structure, positively affected the development of close relationships among family members, and improved the treatment of mental illness in middle-aged and elderly people [18,19]. Increased intragenerational mobility, meanwhile, has been found to benefit Chinese citizens’ physical and mental wellbeing. Upward mobility can benefit the health of low-income groups, as evidenced by a lower risk of depression [20]. At the same time, people with high income mobility are more likely to have access to more equitable economic opportunities [21]. In this case, external environmental limitations on an individual’s income status are weakened, while the effect of family resource transmission on children’s income status decreases [22,23]. This can strengthen Chinese people’s trust in society and thereby improve overall harmony and stability.

Some scholars have recently attempted to examine the role of educational homogamy in terms of intergenerational mobility. Using the 2010–2018 China Family Panel Studies (CFPS) micro-survey data, Tan et al. [24] found that educational homogamy exacerbates the solidifying of intergenerational income, which widens the intergenerational income gap. There is, however, an unfortunate lack of specialized studies on the relationship between educational homogamy and income mobility. Scholars have also mostly examined mobility in terms of intergenerational mobility, while ignoring the existence of intragenerational mobility. Inter- and intragenerational mobility are important parts of overall income mobility, one cannot replace the other [25]. For this reason, this paper combines inter- and intragenerational income mobility to measure the income mobility of China’s residents comprehensively, while examining the impact of homogamy on income mobility to explore the cause of this income gap.

This study thus designed two data samples, one each for intergenerational mobility and intragenerational mobility. The former is the income correspondence sample of a parent and child in the same household, whereas the latter is the sample tracking income in the same household over multiple years. After controlling for life-cycle and temporary fluctuation biases in the analysis, this paper examines the role of educational assortative mating among Chinese residents in inter- and intragenerational mobility. This paper makes the following three contributions. First, it examines the impact of educational homogamy on social fairness from the perspective of income mobility, filling the gap in traditional research on this relationship. Second, it combines inter- and intragenerational methodologies to consider the level of income mobility among Chinese residents to yield more comprehensive analytical results.Third, separate studies are conducted on different income classes to reveal the key factors constraining the upward mobility of low-income groups, which in turn provides ideas for new solutions to reduce this income gap and realize social equity.

## 2. Theoretical analysis and research hypothesis

### 2.1 Educational homogamy and income mobility

Marriage is one way to start a family, and the mode of matching affects people’s living conditions and social resource allocation. Marriage involves exchanging valuable resources to maximize the utility of both parties [26]. In China, the acceleration of modernization, extension of primary education, and improvement of educational access for women not only offer the prospect of educational homogamy but also produce social stratification and change, resulting in new types of social inequality [27].

Educational homogamy can exacerbate imbalances in the allocation of educational resources among different families and pose challenges to solidifying income distribution among families with varying educational levels [28]. Educational disparities are often caused by unequal family education resources, and educational homogamy involves the reintegration of families with comparable educational resources [29]. It is difficult for family members to improve their income status by boosting their educational levels since families based on low-education-homogeneous marriages have amassed excessive subpar educational resources over time. At the same time, the failure of family cultural capital creation is linked to parents’ lack of educational resources. Children are more likely to experience “educational poverty” and “income poverty” over generations as a result of inadequate support for educational resources and cultural capital development [30]. Meanwhile, by assembling a wealth of beneficial educational resources, families based on high-education-homogeneous matching can sustain higher income levels and achieve the transmission of economic status both within and across generations.

Homogeneous schooling reinforces the benefits and drawbacks of various income levels and widens the income disparities between them. Marriage matching modifies income distribution by altering the share of various family types and the division of labor between spouses in the household. Educational homogamy symbolizes the pursuit of economic interests among local people. The possibility of wealth accumulation is increased by this marriage-matching mechanism, expanding income disparities across social strata [31,32]. As the economic and social roles of education continue to grow, people’s educational attainment becomes an important indicator of their socioeconomic status. Thus, “intermarriage within class” in contemporary culture is essentially what educational homogamy is, although it has evolved over time. Under the paradigm of educational homogamy, people tend to choose mates who share or nearly share their own traits, further separating income groups [33]. Educational homogamy has exacerbated the Matthew effect in the job market, raising barriers between different classes of people and lowering income mobility in society [34,35]. Based on the above, the following is proposed:

H1: Educational homogamy reduces intergenerational and intragenerational mobility.

### 2.2 Mechanism of family educational investment

In modern societal growth, improving education levels can help individuals establish future economic advantages [36]. Education accelerates the growth of human capital (e.g., individual knowledge and skill levels), improves people’s production capacity, and enables workers to obtain higher returns and income status in the labor market, all of which affect people’s educational investment decisions [37]. When making educational investment choices, people will consider various criteria, such as education cost, projected returns, and enrollment risk [38]. In recent years, as China’s economy and society have developed and education has expanded, the cost of education and the risk of additional education have been greatly lowered. At the same time, the skill premium for high-skilled workers in the labor market continues to rise, implying that the expected return on educational investment has increased, making Chinese people confident about the return on educational investment [39]. As a result, an increasing number of Chinese families aim to achieve higher levels of education through increased educational investment, helping them to better compete in the labor market.

Education is critical to the transmission of economic status in the income mobility process. High-quality education is essential for improving people’s lives and achieving long-term societal development. It narrows the gap in development opportunities between income groups and is a valuable tool for promoting income mobility and social fairness [40]. This could explain why people are so enthusiastic about investing in education. For high-income families, educational investment is a way to maintain the family’s social status. For low-income households, meanwhile, investing in education is a way to move ahead. However, the prevalence of educational homogamy reduces the importance of educational investment in income accumulation, thus lowering income mobility. The reasons are as follows.

**Fig 1:**
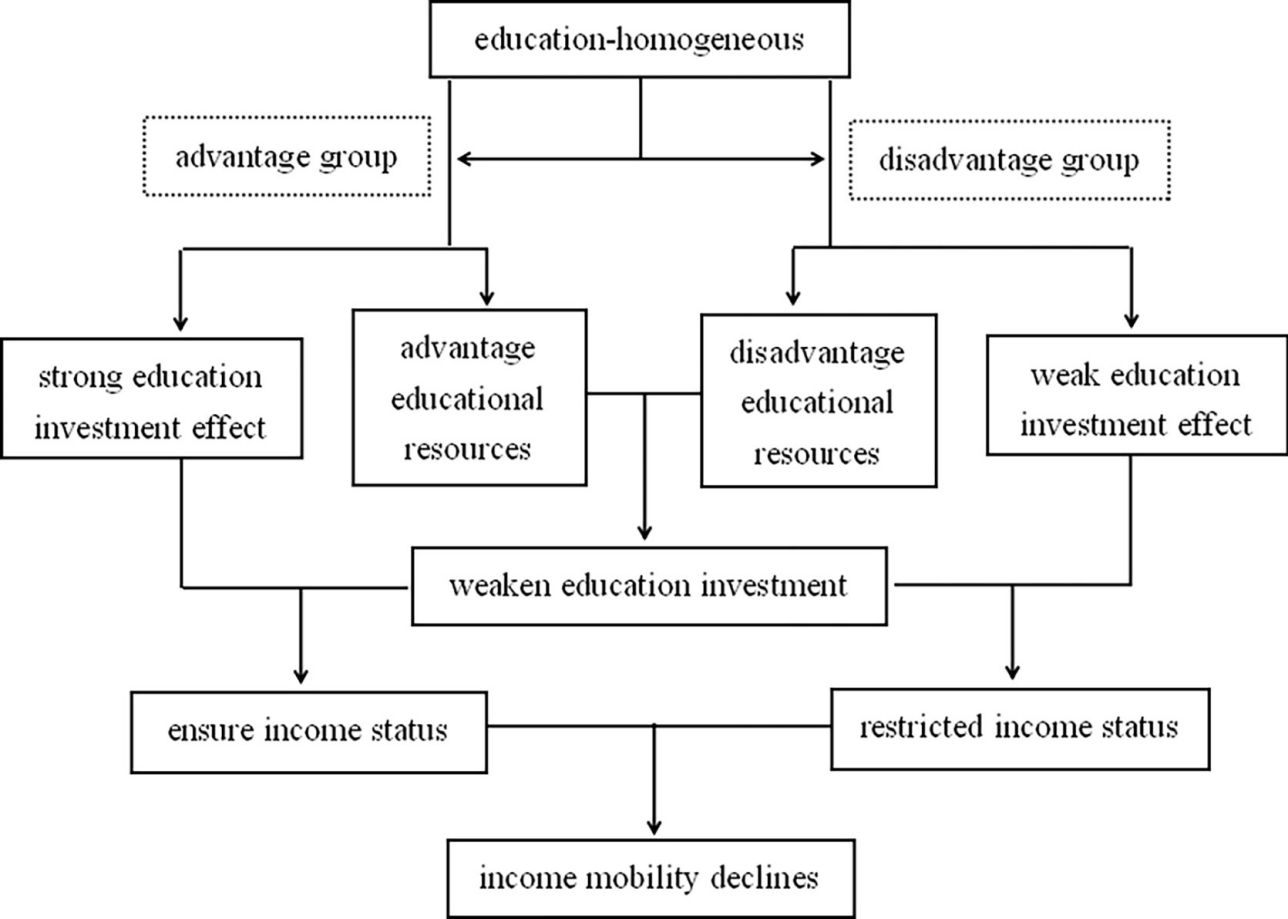
Theoretical framework.

First, educational homogamy causes disparities in the effectiveness of educational investment across families of varying educational levels. When there is educational homogeneity in marriage matching, people with similar cultural tastes, behavioral preferences, and development goals form family cooperation alliances, resulting in disparities in the outcomes of various family educational investments [41]. Families with high levels of education have more expertise and benefits in terms of the intensity and operation of educational investment, allowing them to gain more significant investment returns [42]. However, there is more blindness in educational investment among families with low education levels, and the investment benefit tends to be insignificant. Second, educational homogamy undermines the function of educational investment in people’s income acquisition by increasing disparities in educational resources across various households, which is also the main reason educational homogamy limits income mobility. Family education resources are the primary means of helping family members improve their education and income levels, which can compensate for individual talent deficiencies and insufficient internal growth capacity. However, educational homogamy exacerbates disparities in the distribution of educational resources across families with varying educational levels and class statuses. Highly educated or upper-class family members tend to occupy more high-quality educational resources, which means they have more educational opportunities and development opportunities. Less educated or lower-class family members have disadvantages in acquiring educational resources and opportunities [43]. This will result in educational investment playing a greatly diminished role in the acquisition of income, reducing the ability of vulnerable groups to close the income gap through educational investment, thereby weakening society’s overall income mobility (Fig 1 depicts this theoretical framework). Based on this analysis, the following are proposed:

H2a: Educational homogamy reduces intergenerational and intragenerational mobility by diminishing the role of educational investment in income acquisition.
H2b: Educational homogamy dramatically limits the role of educational investment in income acquisition among low-educated or low-income households, reducing both intergenerational and intragenerational mobility for these groups.

## 3. Model design and variable description

### 3.1. Model design

A benchmark regression model of the relationship between educational homogamy patterns and intergenerational mobility was constructed:

$$rank_{i,s}=\alpha +\beta_{0}rank_{i,f}+\beta_{1}match_{i}+\beta_{2}match_{i}\times rank_{i,f}+\gamma X_{i}+\varepsilon_{i}$$ (1)

Where *i* is family, *f* is the parent’s generation, and *S* is the child’s generation; *rank*_*i*,*f*_ denotes the income quartile of the parent’s generation, and *rank*_*i*,*s*_ denotes the income quartile of the child’s generation. Here, *β*_0_ indicates the intergenerational mobility rate—that is, the correlation between the income quartile of the child and that of the parent. A larger *β*_0_ value means a stronger correlation between the income status of the child and the parent but a lower level of intergenerational mobility. A smaller *β*_0_ value means a smaller correlation between the income status of the child and the parent but a higher level of intergenerational mobility, *β*_0_ takes a value between 0 and 1. Variable *match*_*i*_ denotes the pattern of educational homogamy for the parents in the family, *β*_1_ indicates the degree of influence of parents’ educational homogeneous marriage on the income status of offspring. The interaction term *match*_*i*_×*rank*_*i*,*f*_ is added as the core explanatory variable, and *β*_2_ denotes the influence of the pattern of educational homogamy on the intergenerational mobility rate. If *β*_2_ is regular, it indicates that parents’ educational homogeneous marriage increases the rate of intergenerational mobility, thereby weakening intergenerational mobility. *X*_*i*_ is the individual control variable, and *ε*_*i*_ is the random error term.

A benchmark model of the relationship between educational homogamy patterns and intragenerational mobility was also constructed:

$$rank_{i,t}=\alpha +\beta_{0}rank_{i,t-1}+\beta_{1}match_{i}+\beta_{2}match_{i}\times rank_{i,t-1}+\gamma X_{i}+\varepsilon_{i}$$ (2)

where *i* is the individual, *rank*_*i*,*t*_ and *rank*_*i*,*t*−1_ denote the individual’s income quartile in the *t* time period and the *t*−1 time period, respectively. Variable *β*_0_ indicates the intragenerational mobility rate—that is, the correlation between the income status of the same individual at different periods; a larger *β*_0_ value means a lower level of intragenerational mobility, while a smaller *β*_0_ value means a higher level of intragenerational mobility, *β*_0_ takes a value between 0 and 1. Variable *match*_*i*_ denotes the pattern of educational homogamy of the couple in the household. Here *match*_*i*_×*rank*_*i*,*t*−1_ is the core explanatory variable, whose coefficient *β*_2_ denotes the influence of educational homogamy on the intragenerational mobility rate. *X*_*i*_ is the household control variable.

In the theoretical analysis, this paper proposes the mechanism of family education investment in the influence of education homogeneous marriage on income mobility. Models (1) and (2) each include variables related to educational investment. The mechanism analysis model is built as follows:

$$rank_{i,s}=\alpha +\beta_{0}rank_{i,f}+\beta_{1}match_{i}+\beta_{2}match_{i}\times rank_{i,f}+\beta_{3}invest_{i}+\beta_{4}match_{i}\times invest_{i}+\gamma X_{i}+\varepsilon_{i}$$ (3)

$$rank_{i,t}=\alpha +\beta_{0}rank_{i,t-1}+\beta_{1}match_{i}+\beta_{2}match_{i}\times rank_{i,t-1}+\beta_{3}invest_{i}+\beta_{4}match_{i}\times invest_{i}+\gamma X_{i}+\varepsilon_{i}$$ (4)

where *invest*_*i*_ represents family education investment. If the effect of the educational homogamy pattern on income mobility is no longer significant after adding the interaction term, it would suggest that the education investment factor can fully explain the role of educational homogamy pattern on income mobility; if the effect of educational homogamy pattern is still significant after adding the interaction term, but the coefficient value changes, then the education investment factor may partially explain the role of the educational homogamy pattern.

### 3.2. Variable declaration

In the analysis of intergenerational mobility, the model variables are:

Explained variable: income quartile of the child’s generation (Rank_s_). All children in the same year are here equally divided into 100 percentiles according to their income levels, from low to high; the income ranking of individuals in the same generation is the child’s income quartile.

Explanatory variables: 1. income quartile of the parent’s generation (Rank_f_). All parents in the same year are here equally divided into 100 percentiles according to their income levels, from low to high; the income ranking of individuals in the same generation is the parent’s income quartile. 2. pattern of educational homogamy for the parents (Match_p_). If the parents’ education levels are the same, it is expressed as one; otherwise, it is expressed as zero. 3. interaction term (Match_p_×Rank_f_). This is the core explanatory variable, expressed as the product of Match_p_ and Rank_f_.

Individual control variables: include control variables for the child’s generation as well as for households. The control variables for the child’s generation include child’s age (CA) and the square of age (CA2); child’s gender (Gender), with 1 for male and 0 for female; registered permanent residence (Registry), with 1 for non-agricultural and 0 for agricultural; marital status (Marriage), with 1 for married and 0 for other statuses; work status (Work), with 1 indicating being a member of the workforce and 0 for other work statuses; insurance status (Insurance), with 1 for being insured and 0 for being uninsured; and health status (Health), with 0 for unhealthy, 1 for average, and 2 for healthy. The household control variables include permanent residence (Residence), with 1 for urban and 0 for rural; father’s age (FA) and age squared (FA2); mother’s age (MA) and age squared (MA2); and number of household members (Size).

Mechanism variable: family education investment (Invest). This paper measures the (in logarithm) of family education training expenditures from 2010 to 2020, and education expenditure for 2012–2020 is adjusted according to the Consumer Price Index of 2010, while the same household’s expenditure on education over multiple years is characterized by the average.

In the analysis of intragenerational mobility, the model variables are:

Explained variable: 2020 income quartile (Rank_2020_). The income of all individuals in 2020 are divided into 100 percentiles, and the individual’s income ranking among all individuals is the income quartile.

Explanatory variables: 1. 2010 income quartile (Rank_2010_). The income of all individuals in 2010 are divided into 100 percentiles, and the individual’s income ranking among all individuals is the income quartile. 2. pattern of educational homogamy for the couples (Match_c_). If the couples’ education levels are the same, it is expressed as one; otherwise, it is expressed as zero. 3. interaction term (Match_c_×Rank_2010_). This is the core explanatory variable, expressed as the product of Match_c_ and Rank_2010_.

Household control variables: include usual residence (Residence), with 1 for urban and 0 for rural; individual age (IA) and age squared (IA2); spouse’s age (SA) and age squared (SA2); and number of household members (Size).

Mechanism variable: family education investment (Invest).

### 3.3. Data source

Because this paper analyzes patterns of educational homogamy and income mobility within Chinese marriages, nationwide microdata are required for couples’ education levels, individual income tracking, and the independent income of parent’s and child’s generations. Among the microdatabases accessible, the CFPS best meets the research requirements. Implemented by the Institute of Social Science Survey (ISSS) at Peking University, the CFPS tracks changes in Chinese residents’ family information over multiple years. The survey is conducted once every two years, covering more than 16,000 households in 25 major provinces (municipalities/autonomous regions) in China. It is nationally representative in terms of detailed information on the income, gender, age, education level, and family relationships of Chinese household residents. Data from six surveys—CFPS 2010, 2012, 2014, 2016, 2018, and 2020—have been used to construct the sample for analysis.

For the intergenerational mobility sample, the total annual income from the CFPS individual database has been used to measure the income levels for the parent’s and child’s generations. Sources of income include employment, self-employment, and agricultural cultivation; only the sample of those with positive income is included for the parent’s generation, this paper requires that income data exist for both the father and mother; the income level of the parent’s generation is measured by the total income of both parents [44]. Given that multigenerational households, as well as multiple children, are common in Chinese families, this paper includes the grandfather, father, and children of the same family in the target sample; the number of parent–child pairs in the family is measured according to the number of child’s generations. The parent–child pairs in the data from the CFPS 2010, 2012, 2014, 2016, 2018, and 2020 are vertically spliced, and the duplicate income values of the same individual in multiple years are deleted to obtain the parent–child pair income data for each family.

This paper uses the per capita net household income from the CFPS household database to measure the intragenerational mobility sample. The total household income data encompass wage income, business income, asset income, transfer income, and other income (gifts and gratuities) for household members; the net household income deducts the production cost of business income on this basis. This paper only includes the sample of individuals with a positive income; 2010 is taken as the base year, which is then horizontally matched with the CFPS 2010, 2012, 2014, 2016, 2018, and 2020 data based on household codes to obtain income tracking data for the same household over multiple years.

In the analysis of income mobility, life-cycle and temporary income biases are common factors leading to estimation bias. To reduce the life-cycle bias, this paper restricts the age of parents and children in the intergenerational mobility sample to 20–65 years old. The age data in the intragenerational mobility sample is measured by the average age of the household members, which is also restricted to the age range of 20–65 years old, which is when individuals tend to have a more stable income level. Individuals’ age and age squared are also added to the regression analysis to further control for life-cycle bias. Regarding the temporary fluctuation of income bias, the income level at a certain period may be disturbed by temporary income, and the income in a single year is not sufficient to characterize the long-term income level, so the average income value in multiple years is more robust [45]. Income values for the parent’s and child’s generations over multiple years are thus averaged in the intergenerational mobility sample. More specifically, the income data for 2010–2012 and 2018–2020 are averaged in the intragenerational mobility sample to represent the income of the household in 2010 and 2020, respectively. The intragenerational mobility rates utilized in the analysis are all estimated and obtained from individuals’ income data in 2010 and 2020. Finally, to ensure comparability across years, the income data in the inter- and intragenerational mobility samples for 2012 and beyond are deflated according to the 2010 Consumer Price Index.

To assess educational homogamy, the sample of couples was limited to ongoing legal marriage, not including cohabitation without legal marriage, divorce, or widowhood. To identify education level, the highest level of education obtained by both spouses was measured at the time of the survey. Drawing on the criteria of the China Education Statistical Yearbook—and taking into account the overall cultural and educational level of Chinese society in the current period—individuals’ educational attainment has also been divided into three levels: primary education (elementary school education level and below), intermediate education (including junior high school, senior high school, junior college, technical school, vocational high school, and other equivalent levels), and advanced education (including college, undergraduate education level, and above).

Individual characteristic variables other than income in both the inter- and intragenerational mobility samples were obtained from the CFPS 2010 Household Database. Table 1 reports the descriptive statistics for the main variables.

**Table 1:** Descriptive statistics of the main variables.

Sample	Variable	Sample size	Mean	Standard deviation	Min	Max
Intergenerational sample	Rank_s_	2458	50.079	29.072	1	100
	Rank_f_	2458	50.376	28.958	1	100
	Match_p_	2458	0.637	0.481	0	1
	Match_p_×Rank_f_	2458	31.697	33.200	0	100
	CA	2458	26.123	4.692	20	44
	Gender	2458	0.664	0.472	0	1
	Registry	2458	0.360	0.480	0	1
	Marriage	2458	0.406	0.491	0	1
	Work	2458	0.820	0.384	0	1
	Insurance	2458	0.831	0.375	0	1
	Health	2458	1.134	0.840	0	2
	Residence	2458	0.537	0.499	0	1
	FA	2458	52.437	5.764	38	65
	MA	2458	50.808	5.651	28	65
	Size	2458	4.525	1.753	1	14
	Invest	1310	7.861	1.171	3.175	11.410
Intragenerational sample	Rank_2020_	5037	50.490	28.869	1	100
	Rank_2010_	5037	50.485	28.869	1	100
	Match_c_	5037	0.622	0.485	0	1
	Match_c_×Rank_2010_	5037	31.083	33.345	0	100
	Residence	5037	0.432	0.495	0	1
	IA	5037	47.171	10.444	20	65
	SA	5037	47.611	10.262	20	65
	Size	5037	4.160	1.893	1	21
	Invest	4651	7.818	1.214	1.649	11.410

## 4. Empirical analysis

### 4.1. Benchmark regression

The influence of individuals’ educational homogamy pattern on income mobility was analyzed using OLS regression methods for cross-sectional data. Table 2 reports the results of the benchmark estimation of the influence of patterns of educational homogamy on intergenerational mobility. As shown, the coefficients of the interaction term effects of educational homogamy pattern and parental income quartile are all significantly positive and statistically significant, which indicates that parents’ educational homogamy enhances intergenerational mobility rate—that is, educational homogamous behavior reduces intergenerational mobility. If the parents in the family have the same level of education, the income status of the children is more likely to be held back by the income status of the parent’s generation. Parents’ educational homogamy thus reduces the fairness of intergenerational income distribution. The educational homogamy of the parent’s generation also has a significant negative effect on the income quartile of the child’s generation, which indicates that parents’ educational homogamy reduces the income quartile of the child’s generation.

**Table 2:** Educational homogamy and intergenerational mobility.

Variable	(1)	(2)	(3)	(4)
Rank_f_	0.417***	0.329***	0.410***	0.319***
	(0.030)	(0.029)	(0.029)	(0.029)
Match_p_	−5.577***	−5.511***	−6.332***	−5.371***
	(2.068)	(1.939)	(2.048)	(1.942)
Match_p_×Rank_f_	0.089**	0.093***	0.098***	0.091***
	(0.037)	(0.035)	(0.036)	(0.034)
CA		8.495***		7.324***
		(1.056)		(1.277)
CA2		−0.135***		−0.115***
		(0.018)		(0.022)
Gender		4.461***		4.435***
		(1.041)		(1.037)
Registry		1.886*		1.104
		(1.100)		(1.195)
Marriage		−0.317		1.339
		(1.166)		(1.231)
Work		13.242***		13.180***
		(1.268)		(1.265)
Insurance		6.067***		6.043***
		(1.318)		(1.322)
Health		−4.734***		−4.433***
		(0.621)		(0.619)
Residence			−0.504	0.302
			(1.100)	(1.119)
FA			4.542**	1.870
			(1.897)	(1.871)
FA2			−0.039**	−0.017
			(0.018)	(0.018)
MA			2.732	0.226
			(1.817)	(1.834)
MA2			−0.021	−0.002
			(0.018)	(0.018)
Size			−1.258***	−1.338***
			(0.318)	(0.325)
Observations	2458	2458	2458	2458
R^2^	0.2245	0.3401	0.2610	0.3459

Note

* p < 0.1,

** p < 0.05,

*** p < 0.01; robust standard errors in parentheses.

Table 3 reports the results of the benchmark estimation of the influence of patterns of educational homogamy on intragenerational mobility. The results reported in Table 3 indicate that the coefficient of the interaction term between the pattern of educational homogamy and the base annual income quartile is significantly positive and statistically significant, which suggests that the behavior of educational homogamy leads to an increase in the intragenerational mobility rate—that is, to a decrease in the level of intragenerational mobility. This implies that, for households in which both spouses have the same level of education, individuals are more likely to experience income status solidification in the long run. Taken together, individuals’ educational homogamy significantly reduces the level of inter- and intragenerational income mobility, and the income statuses of both the parent’s generation and the child’s generation are more likely to remain at similar levels.

**Table 3:** Educational homogamy and intragenerational mobility.

Variable	(1)	(2)	(3)	(4)
Rank_2010_	0.466***	0.386***	0.417***	0.360***
	(0.012)	(0.013)	(0.020)	(0.020)
Match_c_			−3.248**	−2.011
			(1.535)	(1.456)
Match_c_×Rank_2010_			0.078***	0.042*
			(0.026)	(0.024)
Residence		11.881***		11.825***
		(0.734)		(0.736)
IA		−4.301***		−4.293***
		(1.043)		(1.045)
IA2		0.082***		0.082***
		(0.018)		(0.018)
SA		0.087		0.086
		(0.104)		(0.104)
SA2		−001***		−0.001***
		(0.001)		(0.001)
Size		−2.597***		−2.578***
		(0.209)		(0.209)
Observations	5037	5037	5037	5037
R^2^	0.2191	0.2995	0.2191	0.3000

Note

* p < 0.1,

** p < 0.05,

*** p < 0.01; robust standard errors in parentheses.

### 4.2. Endogeneity test

To eliminate endogeneity issues, a number of data treatments were performed in the underlying regressions. All income values for individuals over multiple years were averaged, while the education homogamy data were measured using a single year value; individual control variables were included in the regressions. Moreover, in the intergenerational mobility analysis, parents’ marital sorting behavior preceded their children’s income acquisition, and in the intragenerational mobility analysis, couples’ marital sorting behavior preceded the income quartiles in the comparison period. All of these treatments could mitigate endogeneity bias due to reverse causation. However, considering the possibility of omitted variables, which may lead to erroneous measurement of the educational homogamy level and thus affect the robustness of the estimation results, the instrumental variables approach was used as an endogeneity treatment.

The 2SLS regression method was used to regress individuals’ years of education as an instrumental variable to measure the pattern of educational homogamy more precisely. If both spouses have the same number of years of education, then homogamous behavior is present. Table 4 reports the estimation results of the second stage, with Columns (1)–(2) showing the estimation results for the effect of patterns of educational homogamy on intergenerational mobility and Columns (3)–(4) showing the estimation results for their effect on intragenerational mobility. The results indicate that, after regression using instrumental variables, the effect of the pattern of educational homogamy on income mobility remains significantly positive, which indicates that educational homogamy reduces the level of income mobility; this is consistent with the benchmark regression results. Additionally, the F-statistic value of the first stage is much greater than 16, which indicates that there is no weak instrumental variable problem. In the instrumental variable method of estimation, the coefficient values are all significantly larger than the OLS regression coefficient values, which indicates that the negative influence of educational homogamy on income mobility has deepened after re-estimation using the instrumental variable for years of education.

**Table 4:** Estimated results of the instrumental variables approach.

Variable	Intergenerational mobility		Intragenerational mobility	
	(1)	(2)	(3)	(4)
Match_p_×Rank_f_	0.284*	0.244*		
	(0.150)	(0.143)		
Match_c_×Rank_2010_			0.342***	0.191**
			(0.093)	(0.086)
Control variables	No	Yes	No	Yes
First-stage F-statistic values	3686.87	1102.33	5679.95	1908.73
Observations	2443	2443	5037	5037
R^2^	0.2224	0.3055	0.2043	0.2960

Note

* p < 0.1,

** p < 0.05,

*** p < 0.01; robust standard errors in parentheses.

### 4.3. Robustness test

This paper examines the robustness of the following four aspects:

First, changing the age range of the sample individuals. Considering the years of education and the age of retirement among Chinese residents, the age of individuals in the inter- and intragenerational mobility samples was further restricted to range 22–60 years old. Individuals belonging to this age group have a higher probability of being in the labor market.

Second, controlling for sample selection bias in the intergenerational mobility analysis. The sample of the parent’s generation and the child’s generation reported in the CFPS database includes only cases of co-residence. Data on fathers’ income were lacking when children had moved out and were living independently. This missing sample may lead to selection bias that would affect the robustness of the estimation results. To solve this problem, drawing on the approach of Guo et al. [46], the intergenerational income sample was reconstructed using the two-sample two-stage least squares (TS2SLS) method to estimate the income data for the parent’s generation. Unlike the traditional two-sample instrumental variable method (TSIV), TS2SLS is more asymptotically efficient for estimating quantities and more robust in sampling. The CFPS intergenerational mobility data were used as the main sample, while the six surveys of the China General Social Survey (CGSS) data from 2010, 2012, 2015, 2017, 2018, and 2021 were used as the auxiliary samples. The time horizon of the CGSS data surveys, which is similar to that of the CFPS data, is in line with the requirements of TS2SLS estimation. The information about the parent’s generation was divided into father and mother, and the income data of the father and mother were estimated separately. First, a model of the income of the parent’s generation was constructed:

$$\ln\;income_{i}^{f}=\alpha +\beta X_{i}+\varepsilon_{i}$$ (5)

where $\ln\;income_{i}^{f}$ is the logarithm of the income of the parent’s generation; *X*_*i*_ is the individual characteristics of the parent’s generation, including age (Age), age squared (Age2), job attributes (Job), and education level (Edu). Job attributes include non-agricultural work and agricultural work, with non-agricultural work set to 1 and agricultural work set to 0; education level includes primary education, intermediate education, and advanced education, with primary education level set to 1, intermediate to 2, and advanced to 3; *ε*_*i*_ is a randomized perturbation term. The regression results are shown in Table 5.

**Table 5:** Estimated results of the parental individual characteristics.

Variable	Father	Mother
Age	0.000	0.000
	(0.900)	(0.506)
Age2	0.000	0.000
	(0.460)	(0.000)
Edu	0.610***	0.587***
	(0.016)	(0.019)
Job	1.034***	0.875***
	(0.024)	(0.032)
_cons	8.333***	8.370***
	(0.041)	(0.040)
Observations	28754	26050
R^2^	0.1883	0.1326

Note

* p < 0.1,

** p < 0.05,

*** p < 0.01; robust standard errors in parentheses.

Next, using the values of the individual coefficients of the parent’s characteristics derived from the estimation in the first step, the data for parent’s characteristics in the main sample were brought into Eq (5) to predict the data on parent’s income. Finally, the parent’s income values from the second step of the estimation were used to re-match the income data for the parent–child pair and perform the second stage of the intergenerational mobility analysis.

Third, Changing the variable for measuring the rate of intragenerational mobility. Here, net assets per capita were used to replace the income variable to re-measure the level of intragenerational mobility among individuals during the period 2010–2020. Assets, viewed as the accumulation and value added to individuals’ long-term income, have better stability and can better reflect the level of an individual’s wealth in the long run.

Fourth, changing the method for measuring the educational homogamy variables. In the basic analysis, the educational homogeneous behavior of individuals at all levels of education was found to have a significant influence on income mobility, and the degree of this impact varied across different levels of education. To further test this conclusion, drawing on the practice of Eika et al. [47], the classification matching method was used to measure the educational homogamy coefficient of couples at all levels of education to assess the level of educational homogamy. The homogamy coefficient of the basic measurement formula is as follows:

$$s\left(e_{h},e_{w}\right)=\frac{pr(e_{h},e_{w})}{pr(E_{h}=e_{h})pr(E_{w}=e_{w})}$$ (6)

where *s*(*e*_*h*_,*e*_*w*_) is the educational homogamy coefficient of the husband and wife, *e*_*h*_ and *e*_*w*_ denote the specific education level of the husband and wife, respectively, while *E*_*h*_ and *E*_*w*_ denote the education level vector of the husband and wife, respectively. *pr*(*e*_*h*_,*e*_*w*_) denotes the specific matching probability when the education level of the husband and wife is *e*_*h*_ and *e*_*w*_, respectively, and *pr*(*E*_*h*_ = *e*_*h*_)*pr*(*E*_*w*_ = *e*_*w*_) is the random matching probability. Considering the possible difference in the marital sorting concepts of couples of different ages, the age factor is added into Eq (6) to yield Eq (7):

$$s\left(e_{h},a_{h},e_{w},a_{w}\right)=\frac{pr(e_{h},a_{h},e_{w},a_{w})}{pr(e_{h}|a_{h})pr(e_{w}|a_{w})pr(a_{h},a_{w})}$$ (7)

where *s*(*e*_*h*_,*a*_*h*_,*e*_*w*_,*a*_*w*_) denote the educational homogamy coefficient when the husband’s education level is *e*_*h*_, his age is *a*_*h*_, the wife’s education level is *e*_*w*_, and her age is *a*_*w*_; *pr*(*e*_*h*_|*a*_*h*_) denotes the probability of *e*_*h*_ when the husband’s age is *a*_*h*_. The weights of the educational homogamy coefficients of the husband and wife at each age are then constructed:

$$w(a_{h},a_{w}|e_{h},e_{w})=pr(a_{h}|e_{h})pr(a_{w}|e_{w})\frac{pr(a_{h},a_{w})}{pr(a_{h})pr(a_{w})}$$ (8)

As a result, the educational homogamy coefficients could be obtained for all levels after being weighted by couples:

$$\bar{s(e_{h},e_{w})}=\sum_{a_{h}}\sum_{a_{w}}\frac{w(a_{h},a_{w}|e_{h},e_{w})}{\sum_{a_{h}}\sum_{a_{w}}w(a_{h},a_{w}|e_{h},e_{w})}s\left(e_{h},a_{h},e_{w},a_{w}\right)$$ (9)

Education level was again divided into three categories: primary, intermediate, and advanced. Age was divided into two categories: below 50 and 50 or above. The income mobility sample was then divided into 12 sub-samples to obtain the coefficient of the educational homogamy of couples. When the value of this coefficient is greater than 1, it indicates that there is significant educational homogamy; if it is less than 1, educational homogamy is not significant. A larger coefficient value indicates more significant behavioral characterization of educational homogamy. Table 6 reports the educational homogamy coefficients for residents of each province.

**Table 6:** Coefficient of educational homogamy, by province.

Region	Intergenerational mobility			Intragenerational mobility		
	Primary education	Intermediate education	Advanced education	Primary education	Intermediate education	Advanced education
Beijing	2.000	0.545	7.536	1.541	0.941	1.217
Tianjin	1.951	0.360	4.356	3.491	1.306	1.975
Hebei	1.326	1.162	35.343	1.471	1.191	5.903
Shanxi	1.599	2.271	6.630	1.527	1.234	4.391
Liaoning	1.487	1.566	14.884	1.586	1.270	7.610
Jilin	1.485	1.172	33.667	1.519	1.320	6.154
Heilongjiang	1.401	1.845	3.474	1.608	1.161	5.228
Shanghai	1.805	3.239	4.210	1.915	1.224	2.488
Jiangsu	1.566	2.337	6.070	1.786	1.182	1.732
Zhejiang	1.298	2.982	5.127	1.642	1.381	3.123
Anhui	1.004	1.079	3.367	1.224	1.360	6.627
Fujian	1.000	0.223	3.710	1.053	0.781	5.257
Jiangxi	1.201	0.859	6.303	1.310	1.504	5.990
Shandong	1.211	1.416	12.494	1.276	1.117	5.590
Henan	1.502	1.398	6.765	1.589	1.292	5.341
Hubei	1.612	4.915	3.200	2.019	1.216	2.570
Hunan	1.877	1.436	8.813	2.590	1.290	3.494
Guangdong	1.282	1.080	10.948	1.394	1.260	6.339
Guangxi	1.324	1.277	35.458	1.223	1.182	6.380
Chongqing	1.262	1.750	38.714	1.442	1.389	8.250
Sichuan	1.199	1.334	26.109	1.363	1.446	7.176
Guizhou	1.091	0.578	23.167	1.133	1.213	9.524
Yunnan	1.014	0.503	33.700	1.163	1.209	9.527
Shaanxi	1.713	2.081	28.435	1.810	1.241	3.847
Gansu	1.163	0.957	19.374	1.256	1.321	6.391

Our robustness analyses were conducted based on these four points, and the estimation results are presented in Table 7. Columns (1)–(3) are robustness analyses of the influence of educational homogamy patterns on intergenerational mobility, and Columns (4)–(6) are robustness analyses of the influence of educational homogamy patterns on intragenerational mobility. Columns (1) and (4) are estimates after controlling for age, Column (2) shows estimates after reconstructing the sample of intergenerational mobility using the TS2SLS method, Column (5) shows the estimates after reconstructing the sample of intragenerational mobility using the value of assets, and Columns (3) and (6) are estimates after replacing the education homogamy variable with the coefficient of educational homogamy.

**Table 7:** Robustness test.

Variable	Intergenerational mobility			Intragenerational mobility		
	(1)	(2)	(3)	(4)	(5)	(6)
	Control age	TS2SLS	Homogamy coefficient	Control age	Assets	Homogamy coefficient
Match_p_×Rank_f_	0.354***	0.051***				
	(0.029)	(0.012)				
Match_c_×Rank_2010_				0.167***	0.173***	
				(0.012)	(0.011)	
Match_p_×Rank_f_ (primary education)			0.190***			
			(0.024)			
Match_p_×Rank_f_ (intermediate education)			0.028**			
			(0.013)			
Match_p_×Rank_f_ (advanced education)			0.001			
			(0.001)			
Match_c_×Rank_2010_ (primary education)						0.054***
						(0.017)
Match_c_×Rank_2010_ (intermediate education)						0.351***
						(0.035)
Match_c_×Rank_2010_ (advanced education)						−0.030
						(0.004)
Control variables	Yes	Yes	Yes	Yes	Yes	Yes
Observations	1820	5604	2458	4324	4453	5037
R^2^	0.2935	0.1765	0.3249	0.2130	0.2275	0.3147

Note

* p < 0.1,

** p < 0.05,

*** p < 0.01; robust standard errors in parentheses.

Overall, after these control adjustments, the effect of the educational homogamy pattern in marriage on income mobility remains positive and statistically significant. Notably, the coefficient of the impact of the educational homogamy pattern increases after further restricting the age range, which shows that the presence of young and old individuals can lead to an underestimation of the negative influence of the educational homogamy pattern on income mobility. After reconstructing the intergenerational mobility sample using the TS2SLS method, the coefficient of the educational homogamy pattern decreases significantly; after reconstructing the intragenerational mobility sample using asset values, the coefficient of the educational homogamy pattern increases significantly. The educational assortative mating behaviors of couples with primary and secondary education levels also have a significant influence on income mobility, which confirms the results of the previous heterogeneity analysis. The results of the robustness analysis in this paper are consistent with those of the benchmark analysis—that is, individuals’ educational assortative mating behavior significantly reduces the level of income mobility.

### 4.4. Heterogeneity analysis

Income levels were once used to divide individuals into different income classes. The diverse implications of residents’ educational homogamy patterns were then investigated for their impact on economic mobility across classes. With reference to the relative standard measure commonly used in academia, 75%–125% of the median individual income was recognized as the middle-income class, those below that standard were identified the low-income class, and those above that standard as the high-income class [48]. Within the intergenerational mobility sample, strata were classified based on the income level of the parent’s generation; within the intragenerational mobility sample, strata were classified based on the income level of the household in 2010. The estimation results are shown in Table 8, where Columns (1)–(3) are the estimation results of the impact of the pattern of educational homogamy on the intergenerational mobility rate within each strata, and Columns (4)–(6) present the results for its effect within each class on intragenerational mobility. The results show that educational homogamy significantly reduces the level of income mobility among both lower- and upper-class individuals, but the estimates are not significant for the middle class. Within the marriages of low-income class individuals, such behavior has the most significant negative effect on income mobility. When both spouses are in an income disadvantageous position, their own income status—as well as the income status of their children—is shackled by their class to a greater extent.

**Table 8:** Heterogeneity analysis: Income classes.

Variable	Intergenerational mobility			Intragenerational mobility		
	(1)	(2)	(3)	(4)	(5)	(6)
	Lower class	Middle class	Upper class	Lower class	Middle class	Upper class
Match_p_×Rank_f_	0.179***	0.404	0.035*			
	(0.057)	(0.358)	(0.020)			
Match_c_×Rank_2010_				0.137***	0.035	0.038***
				(0.050)	(0.027)	(0.013)
Control variables	Yes	Yes	Yes	Yes	Yes	Yes
Observations	971	536	951	1778	1399	1860
R^2^	0.1499	0.2575	0.1778	0.1010	0.0964	0.1846

Note

* p < 0.1,

** p < 0.05,

*** p < 0.01; robust standard errors in parentheses.

The educational assortative mating behaviors of individuals were further classified into primary, intermediate, and advanced educational homogamy based on the different levels of education to represent the homogamous behaviors of both husband and wife in terms of primary, intermediate, and advanced education levels. This allowed examination of the effect of different educational homogamous behaviors on income mobility, as shown in Table 9. Intergenerational mobility decreases when both parents are at primary and intermediate levels of education, and the effect on intergenerational mobility is most obvious when both parents are at a primary level of education, and insignificant when both parents are at an advanced level of education. Educational homogamy among couples at all levels of education reduces intragenerational mobility, with the highest effect at the intermediate level, the second highest at the primary level, and the lowest at the advanced level. When couples are at a lower income or education level, there is a more pronounced negative relationship between educational homogamy and marital income mobility.

**Table 9:** Heterogeneity analysis: Educational homogamy.

Variable	Intergenerational mobility			Intragenerational mobility		
	(1)	(2)	(3)	(4)	(5)	(6)
	Primary education	Intermediate education	Advanced education	Primary education	Intermediate education	Advanced education
Match_p_×Rank_f_	0.466***	0.347***	−0.758			
	(0.037)	(0.041)	(0.826)			
Match_c_×Rank_2010_				0.330***	0.341***	0.293***
				(0.028)	(0.025)	(0.058)
Control variables	Yes	Yes	Yes	Yes	Yes	Yes
Observations	817	713	35	1364	1520	247
R^2^	0.3271	0.3717	0.4512	0.1850	0.2962	0.4405

Note

* p < 0.1,

** p < 0.05,

*** p < 0.01; robust standard errors in parentheses.

### 4.5. Mechanism analysis

Table 10 reports the role of the educational investment mechanism in income mobility, with Columns (1)–(2) reporting the role of this mechanism in intergenerational mobility. The effect of parents’ educational investment behavior on children’s income quartile is positive and significant at the 1% level, which indicates that educational investment contributes to a rise in children’s income status. After adding the interaction term between educational investment and the educational homogamy pattern, the coefficient of the interaction term between the quartile of the parent’s generation and the educational homogamy pattern is still significant at the 1% level, which indicates that individuals’ educational investment can partially explain the role of educational homogamy as an influence on intergenerational mobility. The coefficient value of the interaction term between the quartile of the parent’s generation and the educational homogamy pattern is also significantly larger, whereas the coefficient of the interaction term between educational investment and the educational homogamy pattern is significantly negative, implying that parents’ educational homogamy weakens the effect of family educational investment on children’s earning status. The earning status of children is thus more subject to the constraint of the family of origin’s class, which leads to a decrease in the level of intergenerational mobility. When there is significant educational homogamy, a higher level of parental education may create more educational resources and development opportunities for the child’s generation, which may then have a higher probability of obtaining the same income status in the future; conversely, a lower level of parental education may limit the educational resources and development opportunities for the child’s generation. Even if the child’s generation consciously invests in education in their growth, it is still difficult for these children to obtain a higher income status.

**Table 10:** Analysis of investment in education mechanism.

Variable	Intergenerational mobility		Intragenerational mobility	
	(1)	(2)	(3)	(4)
Invest	4.390***	2.566***	7.365***	6.212***
	(0.645)	(0.626)	(0.324)	(0.319)
Match_p_×Rank_f_	0.498***	0.374***		
	(0.032)	(0.034)		
Match_c_×Rank_2010_			0.459***	0.362***
			(0.016)	(0.016)
Match×Invest	−3.316***	−2.571***	−2.757***	−2.246***
	(0.279)	(0.290)	(0.146)	(0.142)
Control variables	No	Yes	No	Yes
Observations	1310	1310	4651	4651
R^2^	0.1645	0.3034	0.2083	0.2990

Note

* p < 0.1,

** p < 0.05,

*** p < 0.01; robust standard errors in parentheses.

Columns (3)–(4) of Table 10 report the role of investment in education in intragenerational mobility. The effect of individuals’ investment in education on the income quartile in the comparative period is significantly positive, which indicates that investment in education favors individuals’ own income status. The coefficient of the interaction term between individuals’ educational investment and the educational homogamy pattern is significantly negative, and the coefficient of the interaction term between the educational homogamy pattern and the base-period income quartile has increased, which suggests that individuals’ educational investment can also partially explain the effect of the educational homogamy pattern on intragenerational mobility. Individuals’ educational homogamy also weakens the effect of educational investment on their own income status, which leads to a decline in intragenerational mobility. Taken together, individuals’ investment in education is beneficial for improving their own income status and that of their children, but this effect is limited by educational homogamy, which makes it difficult for individuals to rely on educational investment to break the shackles of educational resources forged by their families of origin. Income solidification appears in the same generation and even between two generations.

### 4.6. Further analysis: Mechanism of low-income class and primary education level residents

The heterogeneity analysis revealed that when individuals’ income status or education level is at a lower level, the weakening effect of their educational homogamy on income mobility is more significant. Individuals with lower levels of education and income are often at a disadvantage in China’s labor market and its income distribution system. Their income mobility is related to the optimization of the size and structure of China’s income groups and the realization of social equity. By focusing on individuals with a lower income and education at the primary level, we continue the examination of the mechanism of investment in education. The estimation results are shown in Table 11. Columns (1)–(2) report the mechanism of investment in education in the intergenerational mobility of individuals with lower income and a primary education level, and Columns (3)–(4) report the impact of this mechanism for intragenerational mobility in this same group. Parents’ educational homogamy was found to limit the role of educational investment in the upgrading of the income status of their children, and the level of income mobility decreases significantly, a characteristic present in low-income parents and those with a primary education level. The lack of quality educational resources and upward career mobility for individuals with low levels of income and education limits their own labor market prospects and those of their children. Although this group has realized the importance of investment in education, their limited investment in human capital has made it difficult to narrow the resource gap between families with different levels of education. The problem is further exacerbated by individuals’ homogamous behavior. Solving the problem of uneven distribution of educational resources that underlies individuals’ homogamy is the key to correctly recognizing the developmental difficulties of low-income groups and breaking down the barriers to their upward mobility.

**Table 11:** Analysis of the low-income class and individuals with primary education level mechanism.

Variable	Intergenerational mobility		Intragenerational mobility	
	(1)	(2)	(3)	(4)
	Low-income class	Primary education	Low-income class	Primary education
Invest	2.103**	9.161***	4.779***	1.577
	(0.942)	(1.071)	(0.501)	(0.992)
Match_p_×Rank_f_	0.587***	0.483***		
	(0.120)	(0.049)		
Match_c_×Rank_2010_			0.251***	0.330***
			(0.037)	(0.028)
Match×Invest	−1.620***	−6.562***	−0.932***	0.001**
	(0.395)	(0.440)	(0.204)	(0.000)
Control variables	Yes	Yes	Yes	Yes
Observations	571	466	1711	1229
R^2^	0.1510	0.3433	0.1572	0.2365

Note

* p < 0.1,

** p < 0.05,

*** p < 0.01; robust standard errors in parentheses.

## 5. Conclusions

In recent years, educational homogamy has become increasingly prevalent in China. Such matching can promote marriage stability and social equality. Based on CFPS data spanning 2010–2020, this study constructs a sample of intergenerational and intragenerational income mobility, investigates the influence of educational homogamy on income mobility, and examines feasible paths for narrowing the income gap in terms of mobility. Educational homogamy is found to be closely associated with improved economic mobility, both intergenerationally and intragenerationally. This is because educational homogamy influences the distribution of educational resources in families with different educational backgrounds, resulting in different families obtaining different educational resources, which affects their intergenerational and intragenerational income status flows. Educational homogamy affects the mobility of low-income and low-education people. Obtaining more high-quality educational resources is crucial for vulnerable populations seeking to advance their income levels. In this sense, when the whole society recognizes the balanced distribution of educational resources, the educational opportunities available to all people will gradually equalize, and the relationship between educational resources and the income status of individuals and children will be greatly narrowed. When individual success is linked to non-environmental qualities such as skill, effort, and even luck, total economic mobility and justice in society will improve [49]. Furthermore, it is no longer necessary to integrate educational resources through marriage matching. People will be more tolerant of their spouses’ educational levels, and the phenomenon of educational homogamy will be diffused, hence increasing income mobility. According to Western modernization theory, the educational homogamy model will grow in an inverted U-shaped pattern as modernization progresses. Individuals’ drive to maintain social and economic standing through marriage weakens as society’s multifaceted mechanisms continue to improve [50].

Based on this, this study makes the following policies and interventions. First, people with strong educational backgrounds are competitive in the labor market. Those with more education are more likely to have good income and social standing. Those with less education find it difficult to secure desirable professions and a stable income. Thus, they prioritize academic qualities in marriage matching. This necessitates breaking the “only education” cycle in the labor market, and educational attainment should not be the sole criterion for people seeking a stable life. On the one hand, we need to improve the employment system; offer flexible multichannel employment platforms; meet the employment needs of people with diverse educational backgrounds, technical levels, and employment needs; and improve laws and regulations to protect the rights and interests of all employed people. On the other hand, the education system should be integrated with labor and employment systems, and higher education and vocational education should be further combined, with the goal of jointly cultivating research and technical talent to meet the labor market’s demand for various skills. Second, China faces an uneven distribution of high-quality educational resources. High-quality educational resources are mainly concentrated in economically developed areas and controlled by a limited group of people. People in impoverished areas do not have access to high-quality educational resources; thus, they place more value on integrating educational resources through marriage. In addition to innate endowments and family educational investment, government education policy affects people’s educational opportunities. The government should focus on equitable opportunity and work to ensure that people have access to balanced educational resources and opportunities. To that end, it is necessary to eliminate educational resource disparities between regions and urban and rural areas, increase investment in human capital in underserved areas, strengthen the construction of basic education facilities, provide multicategory and multilevel educational support for people according to region, and provide more equitable educational starting points and development opportunities for people in various regions. Specifically, we need to further promote rural revitalization, improve urban–rural integration, improve the equalization of basic public services in urban and rural areas, and help rural areas achieve modern living conditions. And, we should establish tailored programs for lower-income people, promote parallel growth in their wealth and economic levels, and keep polarization from becoming worse. Meanwhile, China should establish a multilevel education system, improve the conditions of rural preschools, and raise basic education levels in rural communities. We should carry out expansion and enlargement projects for rural schools, improve the development of high-quality education, equalize resource allocation, and offer external help to low-income communities.

Many recent studies have investigated the relationship between educational homogamy and social inequality, both in China and abroad. Researchers have examined the paths and solutions from many angles, providing a good reference for this study. Different from previous research, this study measures absolute inequality in terms of income mobility rather than the social income gap. The goal is to provide a vertical adjustment method to reduce inequality once it has been established. This work develops an analysis model that includes intergenerational and intragenerational mobility, as well as a more detailed explanation of the specific path of educational homogamy and its effect on social inequality, filling a gap in the research. This study finds that the difference in educational resources between families with different educational levels and income levels is the root cause of the decrease in social mobility and the aggravation of social inequality; educational homogamy exacerbates this problem. Currently, Chinese families face a significant imbalance in the allocation of educational resources. In this circumstance, educational investment at the household level plays a limited role, and it is still necessary to rely on the state to allocate educational resources in a balanced manner to ensure economic justice and equitable educational opportunities. In addition to China, many other countries face an uneven distribution of educational resources. Even in some industrialized countries, educational resources are controlled by elite groups, which reduces social mobility [51]. Drawing on China’s development experience, such nations can implement fairer education and public service policies to attain equity in education and development opportunities based on their own circumstances.

This study has certain limitations. The availability of survey data limits the database; the sample survey period and sample size need to be enlarged. With the ongoing enrichment and development of survey data in China, the study of intragenerational flow can be improved in the future. Future work can continue to investigate the implications of the Chinese government’s recent education initiatives for addressing educational disparity and social equity. Meanwhile, cross-country and Chinese data can be integrated for comparative research to better understand the relationship between educational homogeneity in China and social inequality.

## Supporting information

S1 File (ZIP)

S2 File (DTA)

S3 File (DTA)
